# Fluorescence-coupled micropipette aspiration assay to examine calcium mobilization caused by red blood cell mechanosensing

**DOI:** 10.1007/s00249-022-01595-z

**Published:** 2022-03-14

**Authors:** Haoqing Wang, Peyman Obeidy, Zihao Wang, Yunduo Zhao, Yao Wang, Qian Peter Su, Charles D. Cox, Lining Arnold Ju

**Affiliations:** 1 School of Biomedical Engineering, Faculty of Engineering, The University of Sydney, Darlington, NSW 2008 Australia; 2 Charles Perkins Centre, The University of Sydney, Camperdown, NSW 2006 Australia; 3 Heart Research Institute, Newtown, NSW 2042 Australia; 4 School of Aerospace, Mechanical and Mechatronic Engineering, Faculty of Engineering, The University of Sydney, Darlington, NSW 2008 Australia; 5 Molecular Cardiology and Biophysics Division, Victor Chang Cardiac Research Institute, Sydney, NSW 2010 Australia; 6 Cellular and Genetic Medicine Unit, School of Medical Sciences, University of New South Wales, Sydney, NSW 2052 Australia; 7 School of Biomedical Engineering, Faculty of Engineering and Information Technology, University of Technology Sydney, Sydney, NSW 2007 Australia; 8 Faculty of Medicine, St. Vincent’s Clinical School, University of New South Wales, Sydney, NSW 2010 Australia

**Keywords:** Calcium, Mechanosensitive ion channels, Micropipette assay, Fluorescent imaging, Mechanosignaling

## Abstract

Mechanical stimuli such as tension, compression, and shear stress play critical roles in the physiological functions of red blood cells (RBCs) and their homeostasis, ATP release, and rheological properties. Intracellular calcium (Ca^2+^) mobilization reflects RBC mechanosensing as they transverse the complex vasculature. Emerging studies have demonstrated the presence of mechanosensitive Ca^2+^ permeable ion channels and their function has been implicated in the regulation of RBC volume and deformability. However, how these mechanoreceptors trigger Ca^2+^ influx and subsequent cellular responses are still unclear. Here, we introduce a fluorescence-coupled micropipette aspiration assay to examine RBC mechanosensing at the single-cell level. To achieve a wide range of cell aspirations, we implemented and compared two negative pressure adjusting apparatuses: a homemade water manometer (− 2.94 to 0 mmH_2_O) and a pneumatic high-speed pressure clamp (− 25 to 0 mmHg). To visualize Ca^2+^ influx, RBCs were pre-loaded with an intensiometric probe Cal-520 AM, then imaged under a confocal microscope with concurrent bright-field and fluorescent imaging at acquisition rates of 10 frames per second. Remarkably, we observed the related changes in intracellular Ca^2+^ levels immediately after aspirating individual RBCs in a pressure-dependent manner. The RBC aspirated by the water manometer only displayed 1.1-fold increase in fluorescence intensity, whereas the RBC aspirated by the pneumatic clamp showed up to threefold increase. These results demonstrated the water manometer as a gentle tool for cell manipulation with minimal pre-activation, while the high-speed pneumatic clamp as a much stronger pressure actuator to examine cell mechanosensing directly. Together, this multimodal platform enables us to precisely control aspiration and membrane tension, and subsequently correlate this with intracellular calcium concentration dynamics in a robust and reproducible manner.

## Introduction

**Fig. 1:**
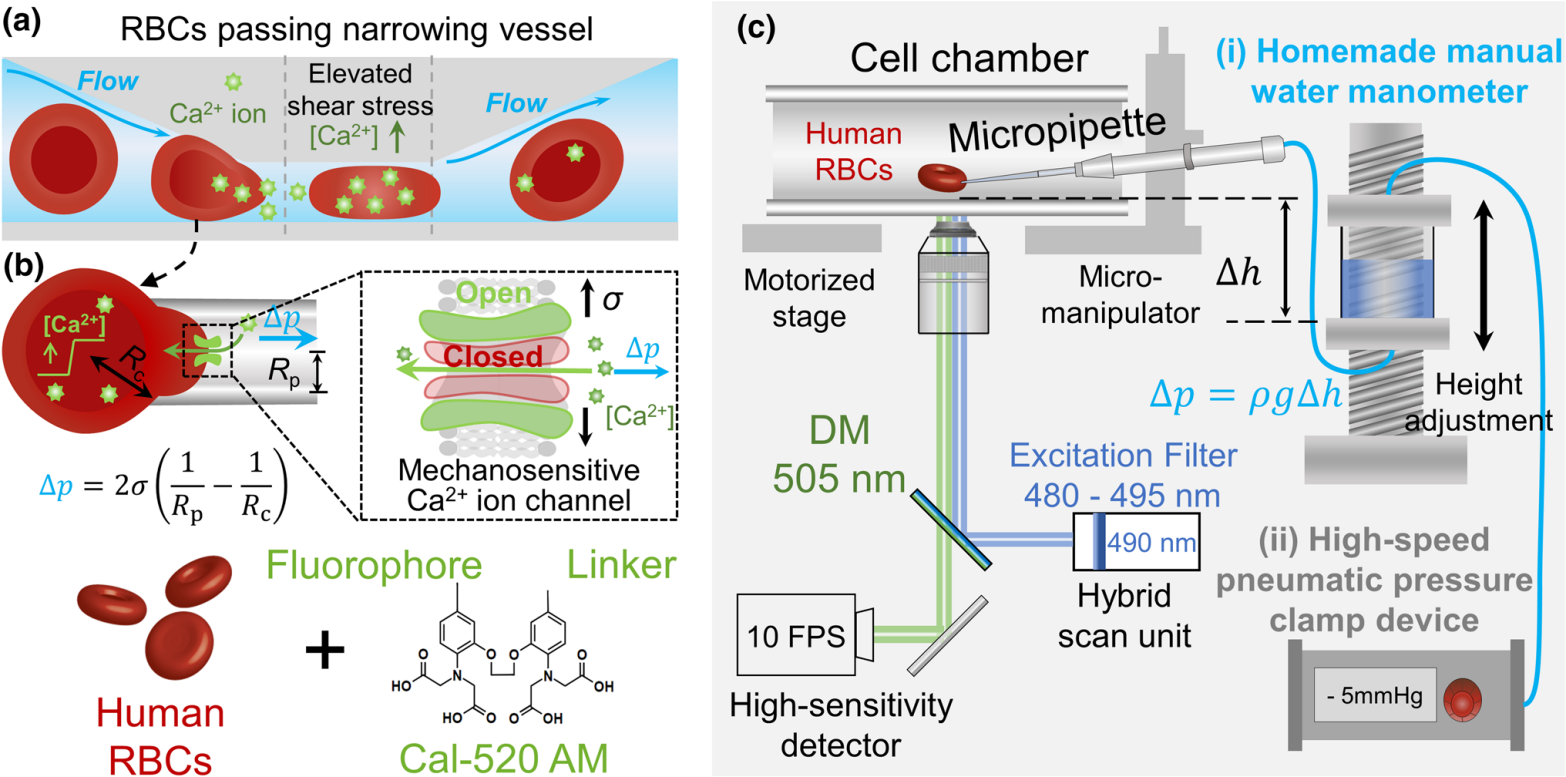
Fluorescence-coupled micropipette aspiration assay for RBCs. **a** Calcium mobilization within RBC under mechanical stimulation. When an RBC passes through a narrowed vessel, elevated shear stress stretches the cell. The induced membrane tension subsequently opens mechanosensitive calcium channels on the RBC membrane and triggers calcium mobilization. **b** Schematic of the fluorescence-coupled RBC micropipette aspiration assay. Top: A borosilicate glass micropipette was fashioned with an open orifice in 0.5 μm radius and used to aspirate a single RBC. The aspiration pressure was precisely controlled by either the water manometer or the pneumatic HSPC. Based on the law of Laplace [Eq. (2)], given the known RBC radius (*R*_c_), the RBC membrane tension (*σ*) can be modulated by adjusting aspiration pressure (*∆p*). Bottom: The RBCs were preloaded with calcium indicator Cal-520 AM which will be excited at the wavelength of 490 nm and emitted at 525 nm. **c** Micropipette system on a confocal microscope. Cal-520 AM loaded RBCs were injected into a homemade cell chamber on the microscope stage. A micropipette was inserted into the cell chamber by operating the micro-manipulator. The RBC aspiration pressure was controlled via a water manometer connecting to the end of the micropipette (apparatus i), or the control panel of the pneumatic HSPC (apparatus ii). The concurrent imaging in both fluorescent (monitor calcium mobilization) and bright-field (monitor cell morphology) lightpaths was achieved at 10 fps. Scanning lasers were emitted from the 488 nm and transmitted light channels in the FV31-HSU hybrid scan unit. The high-sensitivity detector acquired images of both channels

Red blood cells (RBCs) possess excellent mechanical versatility and deformability which enables them to pass through the vessels of the microcirculation and deliver oxygen between blood and tissues. During their life cycles, RBCs (7.5–8.7 μm in diameter) (Diez-Silva et al. 2010) can traverse the circulatory system thousands of times through an extensive network of capillaries (~ 2.5 μm) (Danielczok et al. 2017) or even incredibly smaller splenic inter-endothelial clefts (0.5–1.0 μm) (Liapis et al. 2002). The tension, compression and shear stress experienced by the RBCs lead to ATP release (Cinar et al. 2015; Sikora et al. 2014), rheological property alteration (Chien 1987), nitro oxide generation (Kuck et al. 2020), accelerated glycolysis (Kuchel et al. 2021; Kuchel and Shishmarev 2017) and RBC volume changes. Many of these processes are regulated by the influx of calcium (Brain et al. 2004). The intracellular calcium concentration of unstimulated RBCs is tightly controlled within the range of 30–90 nM (Bogdanova et al. 2013), while the calcium concentration in blood plasma is approximately 1.8 mM (20,000-fold). Increasing evidence has demonstrated that mechanosensitive Ca^2+^ permeable ion channels expressed on the RBC membranes are key transducers of mechanical stimuli, triggering calcium influx that subsequently regulates the volume of RBCs through the Ca^2+^-activated K^+^ channel K_Ca3.1_ (Fig. 1a). To this end, dysregulation of RBC mechanosensing in channelopathies such as hereditary xerocytosis leads to increased intracellular calcium concentration, therefore, dehydrating the cell and accelerating RBC clearance from the bloodstream (Albuisson et al. 2013; Glogowska et al. 2015). However, the cellular events of how RBCs and other mammalian cell types sense mechanical forces and trigger signal transduction are still incompletely understood. To examine the calcium mobilization under controlled mechanical forces, a single-cell platform that can concurrently apply mechanical modulation and visualize calcium mobilization using fluorescent indicators is in great demand.

In the past decades, micropipette aspiration assays have been widely used to mechanically modulate living cells for in-vitro fertilization (Hiraoka and Kitamura 2015; Temple-Smith et al. 1985), gene editing (Rasys et al. 2019; Shao et al. 2014), and characterization of cellular mechanical properties, such as stiffness, viscoelastic modules and membrane tension (Gonzalez-Bermudez et al. 2019; Mierke 2021). With a fashioned open orifice, borosilicate micropipettes integrated with the water manometer can aspirate target live cells in a stepwise manner (Fig. 1b, c):

1 $$\Delta p=\rho g\Delta h$$

where *∆h* (Table 1) is the fluid level difference between the micropipette tip and the reservoir (Fig. 1c, apparatus i). Such controlled aspiration (*∆p*) lends a major advantage to micropipette-based methods that minimize cell pre-activation. This is particularly critical for studies on primary cells, such as platelets (Ju et al. 2016) and T-cells (Liu et al. 2014) which are naturally mechanosensitive. With known radii of the micropipette opening (*R*_p_) and the target cell (*R*_c_) (Table 1), the membrane tension (*σ*) of the aspirated cell can be determined using the law of Laplace (Fig. 1b, top):

**Table 1:** Main parameters for fluorescence-coupled micropipette aspiration assays

Symbol	Definition	Representative value(s)
*R*_p_	Radius of the micropipette orifice	~ 0.5 μm
*R*_c_	Radius of the aspirated RBC	1.5–3.5 μm
∆*h*	Fluid level difference between micropipette and surface level inside reservoir	0.01–40 mm
*∆p*	Aspiration pressure applied by the manual water manometer or the HSPC	− 2.94–0 mmHg (water manometer) − 25–0 mmHg (HSPC)
*F*^p^_max_	Population average maximum fluorescence intensity amongst RBC when 10 μM A23187 + 10 mM CaCl_2_ is added	757 (a.u.)
*F*^s^_max_	Maximum fluorescence intensity of a single aspirated cell after 10 μM A23187 + 10 mM CaCl_2_ is added	821 (a.u.)
*F*^p^_min_	Population average minimum fluorescence intensity amongst RBC when 50 μM BAPTA AM + 10 mM EGTA is added	126 (a.u.)
*F*^s^_min_	Minimum fluorescence intensity of a single aspirated RBC after 50 μM BAPTA AM + 10 mM EGTA is added	129 (a.u.)
*F*^p^_rest_	Average fluorescence intensity of RBC population at the resting state	292 (a.u.)
*F*^s^_rest_	Fluorescence intensity of a single RBC at the resting state	185 (a.u.)
*K*_d_^cal−520^ (Lock et al. 2015)	Dissociation constant of Cal-520 AM when binding to calcium ions	320 nM
[Ca^2+^]_rest_ (Bogdanova et al. 2013)	Intracellular calcium concentration of resting RBCs	30–90 nM

*R*_p_ and *R*_c_ were selected to suit fluorescence-coupled micropipette aspiration assay. Fluorescence intensity was measured either on the RBCs population (*F*^p^_rest_, *F*^p^_max,_ *F*^p^_min_) or the single RBC (*F*^s^_rest_, *F*^s^_max_, and *F*^s^_min_), respectively

2 $$\Delta p=2\sigma \left(\frac{1}{{R}_{\mathrm{p}}}-\frac{1}{{R}_{\mathrm{c}}}\right).$$

Moreover, the pneumatic high-speed pressure clamp (HSPC) device has become a popular assessory tool for patch clamping experiments (Cahalan et al. 2015; Coste et al. 2012; Fogerty et al. 2019; Xue et al. 2020). This is because the pneumatic HSPC is able to apply a high magnitude (*∆p* = − 200–0 mmHg) and steady (± 1 mmHg) pressure clamp to the aspirated cell rapidly (0 to − 100 mmHg in 1 ms). More recently, micropipette aspiration assays have been combined with fluorescent imaging which enables numerous live-cell mechanobiology studies, including nuclear remodeling (Swift et al. 2013), ion channel mechanogating (Cox et al. 2016), and receptor-mediated mechanosensing (Chen et al. 2015, 2019; Liu et al. 2014). Such instrumental integration provided a direct approach to visualize single-cell mechanosensing (Wang et al. 2022b). Nevertheless, there is lack of technical study to head-to-head benchmark the efficacy of water vs. pneumatic apparatus and their distinct mechanical profiles of aspiration on cell mechanosensing (cf. Figure 1c, apparatus i vs. ii).

Hereby, we implemented both manual water manometer and the pneumatic HSPC for single-cell aspiration. We combined micropipette aspiration assays with confocal microscopy, where we used the intensiometric calcium indicator Cal-520 AM (Fig. 1b, bottom) to visualize the calcium mobilization of human RBCs in real time (Fig. 1c). We also determined the dynamic range of the Ca^2+^ imaging system with the Cal-520 AM loaded RBCs using calcium ionophore (i.e. A23187) and calcium chelators (i.e. BAPTA AM and EGTA), which were sequentially added to the experimental chambers. Taken together, this method and experimental setup enabled us to accurately calculate the intracellular calcium concentration under controlled mechanical aspiration.

## Materials and methods

### Reagents

Cal-520 AM (Abcam, Cambridge, UK) was dissolved in dimethyl sulfoxide (DMSO) (2.5 mM) and stored at − 30 °C. Calcium ionophore (5 mM) (A23187; Sigma-Aldrich, US) was dissolved in DMSO and stored at − 30 ℃. BAPTA AM (Thermo Fisher Scientific, Massachusetts, USA) (50 mM) in DMSO, was stored at − 30 °C. EGTA dissolved in Milli-Q water (0.5 M, pH 8) was stored at room temperature with KOH (1:3 w/w). Tyrode’s buffer (12 mM NaHCO_3_, 10 mM HEPES, 0.137 M NaCl, 2.7 mM KCl, 5.5 mM D-glucose, pH 7.2) supplemented with 1 mM CaCl_2_ was prepared and stored at room temperature. Carbonate/bicarbonate buffer (2.1 g Na_2_CO_3_, 2.65 g NaHCO_3_ in 250 mL H_2_O, pH 8.5) was stored at − 4 °C fridge. Clexane (10,000 U/mL, Sigma-Aldrich, St. Louis, MO) was stored at room temperature.

### Human RBC isolation and loading with calcium indicators

All procedures involving the collection of human blood from healthy volunteers were approved by the Human Research Ethics Committee of the University of Sydney (HREC 2014/244). RBCs were isolated from the whole blood as previously described (Chen et al. 2015; Ju et al. 2017; Passam et al. 2018). Briefly, the whole blood was drawn and transferred into a 15 mL tube pre-loaded with 1:200 Clexane. Then 5 μL blood was diluted into carbonate/bicarbonate buffer (1 mL) and centrifuged 900×*g* for 1 min at room temperature. After that, the supernatant was discarded. The washing step was then conducted once with carbonate/bicarbonate buffer and once with Tyrode’s buffer. Finally, the collected RBCs were stored in 200 μL of Tyrode’s buffer at 4 °C.

Isolated RBCs were labeled with 10 μM Cal-520 AM probe for 45 min incubation at room temperature on a vertical spinner. The labeled RBCs were further diluted with a 1:100 ratio in Tyrode’s buffer for experimental use.

### Micropipette fabrication

A borosilicate glass capillary tube (G-1; Narishige International, USA) was mounted on the micropipette puller (model P-1000; Sutter Instruments Co., Novato, CA). Two close-end micropipettes would then be fabricated by the pre-set pulling function in the micropipette puller (Heat: 505; Pull: 150; Velocity: 75; Time: 200; Pressure: 400; Ramp 500). Tips of micropipettes were cut and fashioned to 1 µm diameters using a MicroForge (Model MF-900; Narishige International, USA) equipped with a 20 × eyepiece. For detailed operation procedures, please refer to the previously published protocols (Ju 2019).

### Homemade height-adjusting water manometer to manually control aspiration pressure

The micropipette holder was sealed and connected to the homemade water manometer implemented with a height adjustment mechanism. First, the micropipette holder was taken off and lowered to under the fluid level of the connected reservoir and allowed water dripping for a few seconds. This step can ensure the absence of air bubble inside the tube connecting the micropipette holder and the water reservoir. Then a fully filled borosilicate micropipette fabricated from the previous section was inserted into the holder tip and the holder screw was tightened (Ju 2019). Finally, due to the tightness of the micropipette–reservoir connection, the aspiration pressure of the borosilicate micropipette was finely controlled by adjusting the height of the connected water reservoir related to the tip of the micropipette in a stepwise manner. To this end, the fluid level in the connected reservoir was crucial to be maintained at a constant level during the aspiration assay. Otherwise, to ensure the accuracy of suction pressure, the zero pressure needs to be immediately re-calibrated once the fluid level changes.

### high-speed pressure clamp with aspiration pressure feedback control

**Fig. 2:**
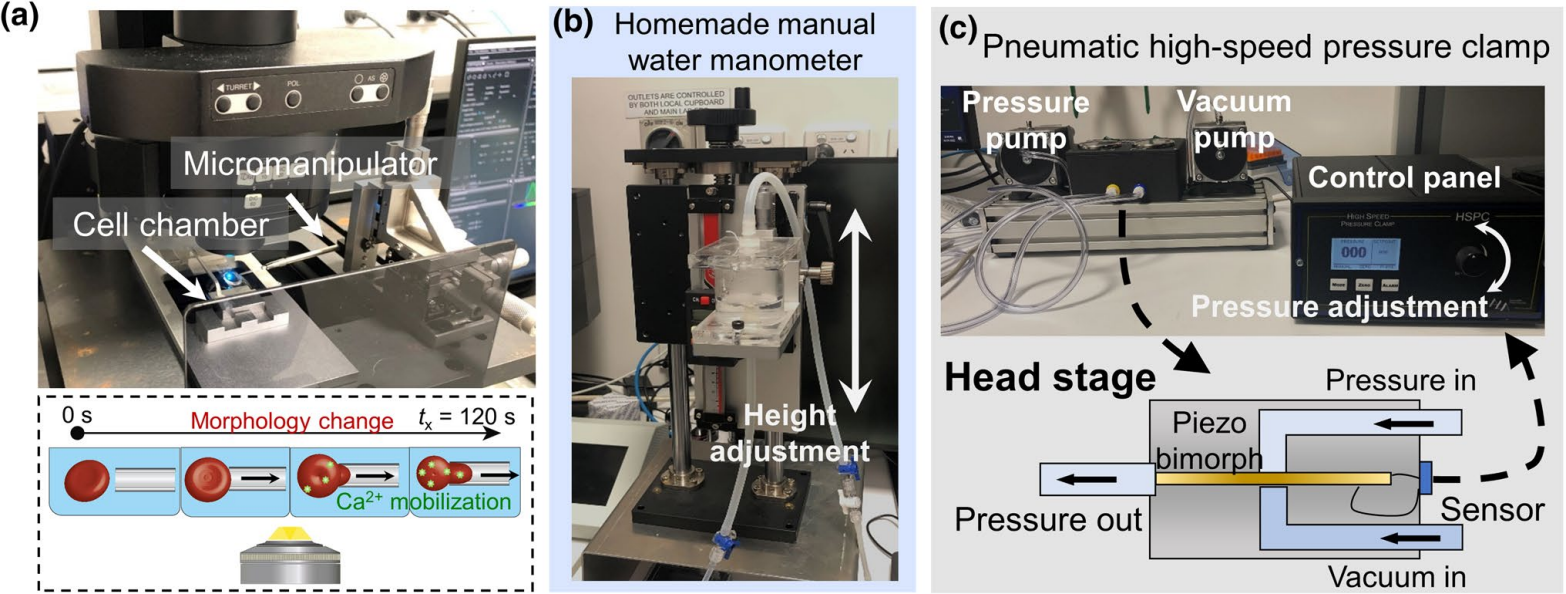
Water manometer and pneumatic HSPC integrated fluorescence-coupled micropipette aspiration setup. **a** Experimental setup of the aspiration assay. Top: a homemade cell chamber with two cut coverslips was placed on the stage of the confocal microscope. After filling the chamber with Tyrode’s buffer and labeled RBCs were injected the micropipette was then inserted into the chamber by turning the micromanipulator. Bottom: schematic of calcium imaging during micropipette aspiration of an RBC (0–120 s). **b** Photo of the homemade water manometer. The pressure was controlled by adjusting the height difference between the fluid level in the reservoir and the tip of the connected micropipette. **c** Pneumatic high-speed pressure clamp device. Top: photo of the pneumatic HSPC’s pressure and vacuum pump. The pressure applied can be adjusted by rotating the knob on the control panel. Bottom: schematic of the head stage. The control panel delivered the voltage to the piezo bimorph in the head stage and mediated the gap between the piezo and the valve seat which determined the output pressure of the device. A sensor was also integrated into the head stage to enable the signal feedback to the control panel

A pneumatic high-speed pressure clamp (HSPC-2-SB; ALA Scientific Instruments, Farmingdale, NY) was also used as an alternative way to provide mechanical stimuli to the aspirated RBC as described elsewhere (Cahalan et al. 2015; Cox et al. 2016). The pressure applied was modified by an electrical control panel (Fig. 2c, top). The output voltage from the control panel was delivered to the head stage that is supplemented by both vacuum and pressure generated by pumps (Fig. 2c, bottom) (Besch et al. 2002). Then the voltage would mediate the spacing between the piezo bimorph and the valve seats inside the head stage which finally control the output pressure at the micropipette tip. A pressure sensor was also integrated inside the head stage to monitor the pressure of the closed system (i.e. micropipette aspiration setup). The system was able to apply a maximum aspiration pressure of − 200 mmHg with a ± 0.5 mmHg resolution and 15 ms response time when a 100 mmHg jump was required. In this study, *∆p* = − 25–0 mmHg aspiration pressure was applied to observe the calcium mobilization inside the RBCs.

### Concurrent imaging of RBC morphology and intracellular calcium

At room temperature, diluted RBC solution (approximately 0.025% hematocrit) was injected into a homemade cell chamber. RBC fluorescence imaging was performed on an Olympus FV3000RS confocal microscope with an IX83 body combined with an FV31-HSU Hybrid Scanning Unit (Tokyo, Japan). Calcium mobilization can then be imaged with laser illumination (Ex 490/Em 525), where 15% power (maximum power: 50 mW) was used in the laser diode. Bright-field imaging was performed simultaneously on the same microscope. Images were illuminated using the silver-coated resonance scanning mirrors at 10 fps and 512 × 512 frame size, controlled with Olympus CellSens Dimension software. Light signals were collected via an Olympus 40 × /0.95 UPLXAPO (Air) objective. Two channels inside the InGaAsP high-sensitivity detector with quantum efficiency > 45% were used to concurrently acquire images of both bright-field light and fluorescent light. The acquisition was performed with averaging every two frames. No significant photobleaching effect was observed during the time interval used in the experiments. All images were then collected with the Olympus FV31S-SW software.

### Intracellular calcium concentration analysis

The correlation between the fluorescence intensity of the calcium dye in the imaging system and labeled cells’ intracellular concentration was investigated by determining the dynamic range of fluorescence intensity. By adding 10 μM A23187 + 10 mM CaCl_2_ or 50 μM BAPTA AM + 10 mM EGTA, the maximum fluorescence intensity, *F*_max_ (a.u.), and minimum fluorescence intensity, *F*_min_ (a.u.) were obtained, respectively. The acquired image was then exported into the analysis software ImageJ (Wayne Rasband, National Institutes of Health) or the commercial software Imaris 9.0.1 (Oxford Instruments, Abingdon, United Kingdom) to perform cell segmentation. To determine the dynamic range based on RBC’s population average, the fluorescence intensity of cells with maximum and minimum fluorescence intensity were then separately plotted with GraphPad Prism v8.0.1 (GraphPad Software Inc., CA, USA) in the form of histograms. Gaussian distributions were fitted to the histograms and the means of distributions were thereafter retrieved as *F*^p^_max_ and *F*^p^_min,_ respectively. Calibration will be used for subsequent intracellular calcium concentration (nM) calculation in Eq. (3). The live time fluorescence intensity of the selected cell can be normalized by dividing the cell’s fluorescence intensity at the resting state. The increase of calcium concentration of the aspirated RBC, *∆*[Ca^2+^] is derived by subtracting the calcium concentration of the RBC in the resting state from the aspirated state. The membrane tension of the aspirated RBC is evaluated by the law of Laplace shown in Eq. (2).

## Result

### Micropipette aspiration of single RBCs on a confocal microscope

To set up micropipette aspiration assays, we first assembled a homemade cell chamber consisting of two metal squares (copper/aluminum) and a handle that links them together. Two pieces of half-cut glass coverslips (40 mm × 11 mm × 0.2 mm) were glued to create a sandwich space with 300 μL Tyrode’s buffer filled up (Fig. 2a, top). After RBCs were injected into the chamber, a fashioned borosilicate micropipette was anchored on the micropipette holder and inserted into the chamber by tuning the micro-manipulator as previously described (Chen et al. 2015; Ju 2019). The micropipette was then approached to grab the target RBC (Fig. 2a, bottom).

**Fig. 3:**
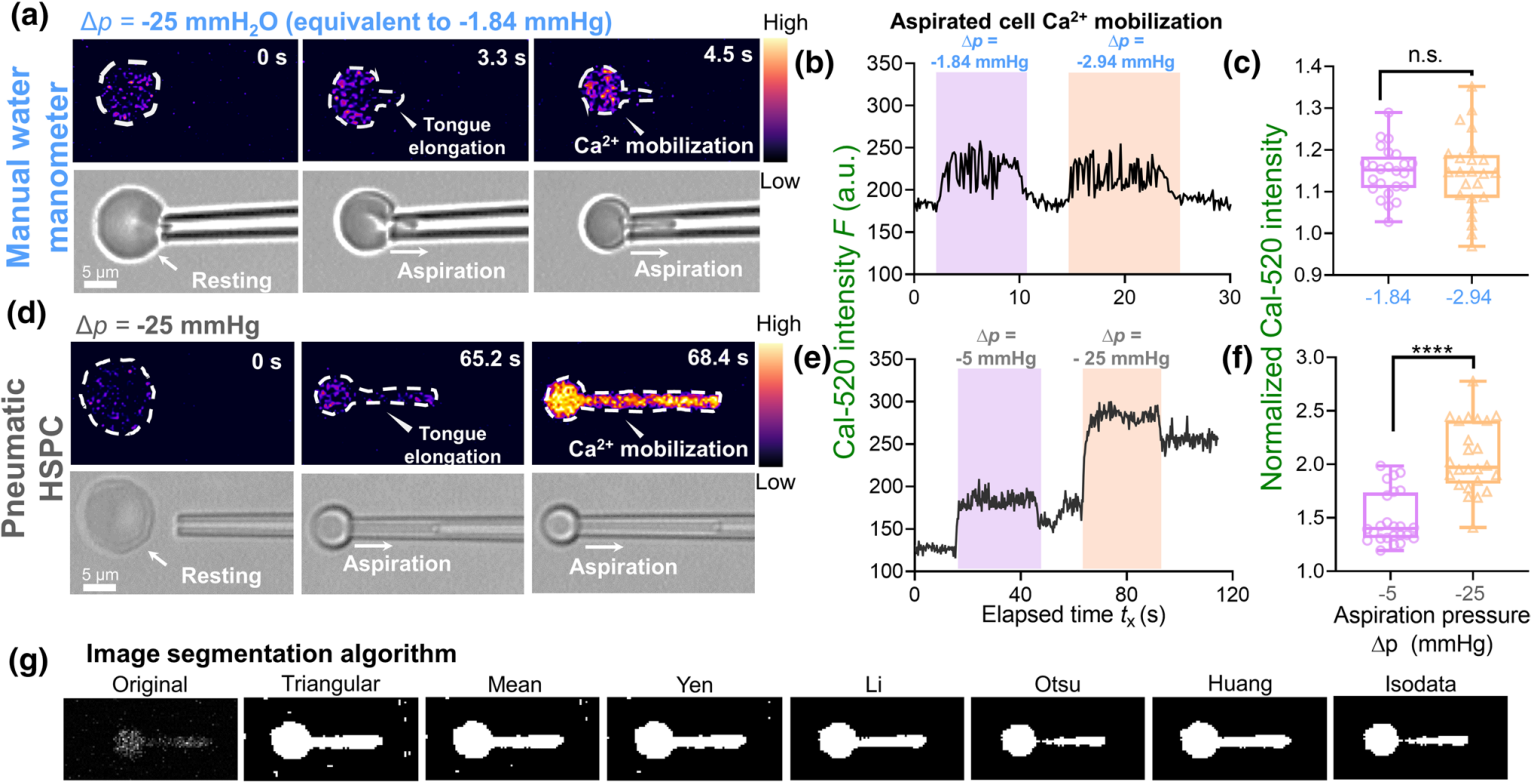
Cell segmentation of aspirated RBC and observation of calcium mobilization under different clamp pressure. **a** RBC was aspirated by the manual water manometer. A slight increase was observed in the Cal-520 intensity after 4.5 s being aspirated by −25 mmH_2_O pressure (equivalent to − 1.84 mmHg). Scale bar = 5 μm. The color bar on the right corresponded to the normalized calcium dependent intensity level. **b** Representative plot of the Cal-520 intensity *F* (a.u.) vs. Elapsed time *t*_x_ (s) trace of the RBC aspirated by a ∆*p* = − 1.84 mmHg and − 2.54 mmHg cycle. The duration of the aspiration is indicated by the shaded background. **c** Box plot of the water manometer aspirated RBC’s normalized Cal-520 intensity under different aspiration pressure. A total number of 25 cells were analyzed in each box plot. *n.s.* not significant, assessed by two-tailed Student *t* test. **d** Snapshots of the pneumatic HSPC aspirated RBC. The RBC was aspirated by a cycle of ∆*p* = − 5 mmHg and − 25 mmHg. A significant Cal-520 increase was observed during the tongue elongation when the RBC was aspirated by the ∆*p* = − 25 mmHg. Scale bar = 5 μm. The color bar on the right corresponded to the normalized calcium dependent intensity level. **e** The representative curve of the Cal-520 intensity (a.u.) subjected to aspiration by a ∆*p* = − 5 mmHg and − 25 mmHg cycle from the pneumatic HSPC. **f** Box plot of the pneumatic HSPC aspirated RBC’s normalized Cal-520 intensity under different aspiration pressure. A total number of 25 cells were analyzed in each box plot. **** = *p* < 0.0001, assessed by two-tailed Student’s *t*-test. **g** RBC image segmentation. Seven thresholding algorithms were benchmarked, where Li, Otsu and Isodata provided excellent signals to noise. We chose Isodata for the subsequent calcium analysis

The cell aspiration was performed using either (i) the homemade water manometer (Fig. 2b) or (ii) the pneumatic HSPC (Fig. 2c). By adjusting the relative height difference with a 0–40 mm travel range (Fig. 2b), the water manometer provided a relatively small pressure range equivalent to ∆*p* = − 2.94–0 mmHg. Notably, based on the previous study (Cahalan et al. 2015), this is below the minimum of − 5 mmHg pressure required to observe a significant calcium influx (cuff Fig. 3b, e). To apply stronger negative pressure to the RBC, we connected a pneumatic HSPC to the top of the reservoir (cf Fig. 1c), which can reproducibly perform aspiration with much stronger negative pressure ∆*p* = − 25–− 5 mmHg (Fig. 2c; cf Fig. 3e).

### Quantitation of intracellular calcium signaling in an aspirated RBC

The intracellular calcium mobilization in RBCs was visualized using the calcium indicator Cal-520 AM (Ex 490/Em 525), which exhibits a significantly improved signal-to-noise ratio and intracellular retention as compared to existing green calcium indicators (e.g., Fluo-3 AM and Fluo-4 AM) (Lock et al. 2015). To achieve a combination of RBC calcium imaging with concomitant monitoring of cell morphology under the aspiration pressure, a confocal microscope was set up using a plan apochromatic dry objective with 40 × magnification and 0.95 numerical aperture (Fig. 1c). A filter cube with a 505 nm dichroic mirror was placed in front of the hybrid scan unit which emitted the 488 nm excitation laser to the calcium dye. Human RBCs were isolated and then incubated with a final concentration of 10 μM Cal-520 AM for 45 min on a vertical spinner at room temperature. This loading concentration was shown to have efficient probe uptake by the cells without functional impairment that might be caused by DMSO as the solvent. By utilizing a silver-coated resonance scanning mirror, the system line scanned the aspirated RBC with both fluorescence and bright-field channels. The signals passed through the dichroic mirror and were acquired by a high-sensitivity detector. This imaging setup was designed to directly quantify the calcium mobilization inside the RBC based on its fluorescence intensity, while observe the morphology change during aspiration (Fig. 2a, bottom).

Since the orifice radius of the micropipette was *R*_p_ =  ~ 0.5 μm, only a small proportion of the RBC can be aspirated into the micropipette, forming an elongating tongue until a steady state is reached (Artmann et al. 1997). As a result, the maximum increase in the Cal-520 intensity observed during aspiration was ~ 50 (a.u.) when the aspiration pressure generated by the water manometer was ∆*p* = − 25 mmH_2_O (equivalent to ∆*p* = − 1.84 mmHg) (Fig. 3a). It reflected a subtle Ca^2+^ influx as compared to the Cal-520 intensity at the resting state *F*_rest_. Moreover, raising the aspiration pressure to the highest level ∆*p* = -40 mmH_2_O (equivalent to ∆*p* = − 2.94 mmHg) failed to further enhance the Ca^2+^ dependent intensity (Fig. 3b). Indeed, both aspiration pressure resulted in 1.1–1.2-fold increase in the aspirated RBC compared to its resting state (Fig. 3c).

To apply a higher pressure, a pneumatic HSPC device connected to the top of the water reservoir was then used. As expected, the Cal-520 intensity raised rapidly in response to both ∆*p* = − 5 mmHg and − 25 mmHg pressure during aspiration (Fig. 3d&e), which is similar to the results obtained by Cahalan et al*.* (Cahalan et al. 2015). Additionally, such increase was pressure-dependent, where the higher pressure led to much greater changes (i.e. ~ twofold under ∆*p* = − 25 mmHg pressure vs. ~ 1.5-fold under ∆*p* = − 5 mmHg; Fig. 3f). It is clear that the pneumatic HSPC is more robust in triggering the Ca^2+^ mobilization than the manual water manometer. Of note, to retrieve the signal only from the RBC without any background noise, seven different image segmentation algorithms were tested: Triangular, Mean, Yen, Li, Otsu, Huang and Isodata (Fig. 3g). Three of them, Li, Otsu and Isodata matched our requirement, and Isodata was chosen as the default thresholding method in this study.

### Determining calcium dynamic range of the RBC labeled with Cal-520 AM

**Fig. 4:**
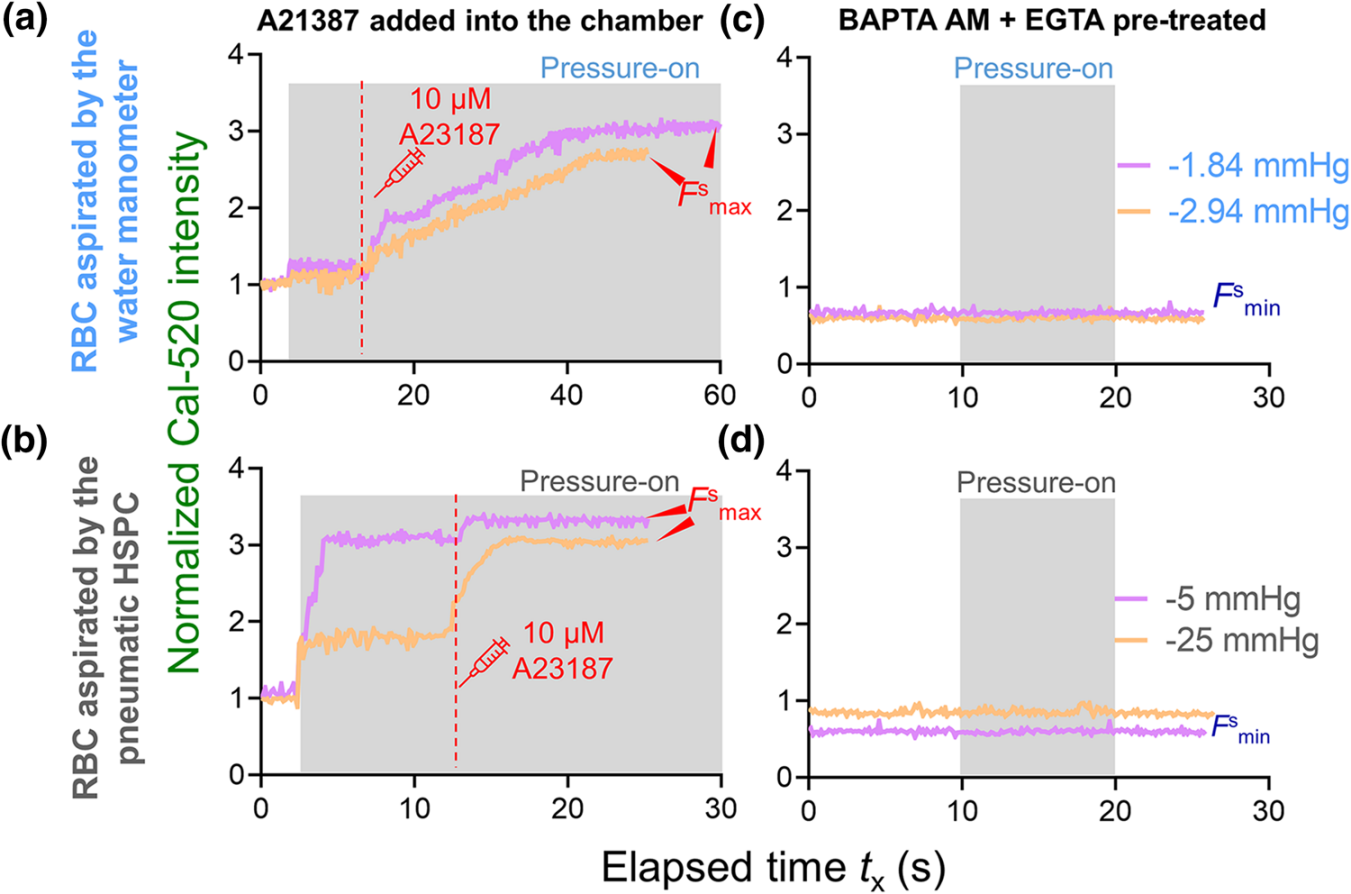
Dynamic range determination on a single aspirated RBC. Representative Ca^2+^ determination curves on cell aspirated by the water manometer (**a, c**) vs. the pneumatic HSPC (**b, d**). **a** and** b** To obtain the maximum fluorescence intensity *F*^s^_max_ (a.u.), 10 μM A23187 + 10 mM CaCl_2_ was added (red dash line) after *t*_x_ = 12 s. **c** and** d** To obtain the minimum fluorescence intensity, *F*^s^_min_ (a.u.), the cell chamber was pretreated with 50 μM BAPTA AM and 10 mM EGTA to chelate all the calcium ions in the intracellular and extracellular space, respectively. The shaded region represented the duration of the pressure applied. All fluorescence was normalized regarding to the aspirated RBC’s fluorescence intensity at the resting state, *F*^s^_rest_

The Cal-520 AM provides a robust, homogeneous fluorescence-based assay tool with a single wavelength for detecting intracellular calcium mobilization (Lock et al. 2015). To determine the Cal-520 dynamic range, we would obtain the single aspirated cell maximum (*F*_max_) and minimum (*F*_min_) of intracellular fluorescent intensities as previously described (Nesbitt et al. 2012), which can be used. The resting intensity (*F*^s^_rest_) was first obtained by imaging one Cal-520 loaded RBC for 50 frames (acquisition rate = 10 fps), followed by another 50 frames acquired after the cell was aspirated. Single RBC was then aspirated using either the water manometer (Fig. 4a) or the pneumatic HSPC (Fig. 4b). To obtain *F*^s^_max_, 10 μM A23187 was added into the cell chamber supplemented with 10 mM CaCl_2_. With both aspiration apparatuses, we observed rapid increase in Ca^2+^ signals until the fluorescent intensity reached a plateau with *F*^s^_max_ being ~ threefold relative to *F*^s^_rest_ (Fig. 4a&b). Notably it took less time for RBCs aspirated by the pneumatic HSPC to reach *F*^s^_max_ (e.g. *t*_x_ = 5–10 s; Fig. 4a), as compared to cells aspirated by the water manometer (e.g. *t*_x_ = 25–30 s; Fig. 4b). Since BAPTA AM and EGTA chelate intracellular and extracellular calcium ions, respectively, we then added 50 μM BAPTA AM + 10 mM EGTA cocktail to obtain the *F*_min_. The Cal-520 intensity of the RBC quickly decayed to the background, which was ~ 0.8-fold of the *F*^s^_rest_ baseline level. As a result, neither water manometer nor pneumatic HSPC induced any Ca^2+^ mobilization inside the RBCs (Fig. 4c&d). Expectantly, both aspiration apparatuses achieved similar outcome of *F*^s^_max_ and *F*^s^_min_; indicating that the Cal-520 dynamic range is independent of the applied mechanical stimuli. This result further demonstrated the validity of the calibrated dynamic range for our cell mechanobiology studies.

**Fig. 5:**
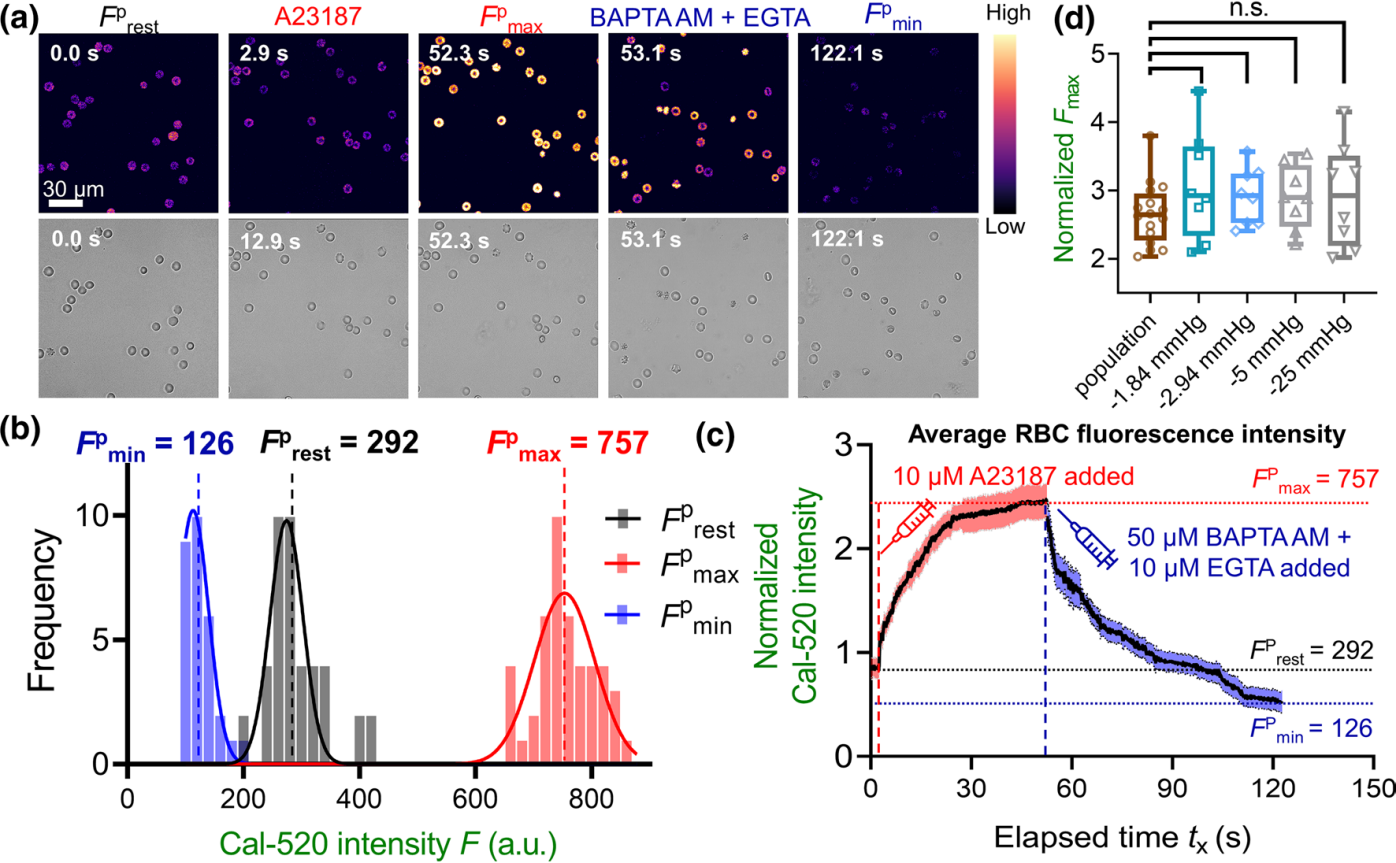
Determination of RBC intracellular Ca^2+^ dynamic ranges in a population assay setup. **a** Concurrent fluorescent (top) and bright-field (bottom) snapshot sequence of RBC population to indicate calcium mobilization and morphology, respectively. RBCs loaded with Cal-520 AM (10 μM) were injected into the cell chamber with the Tyrode buffer. After all cells were settled at the bottom, resting intensity *F*^p^_rest_ = 292 were capture. After *t*_x_ = 2.9 s, 10 μM A23187 + 10 mM CaCl_2_ was added into the chamber and the maximum Cal-520 intensity *F*^p^_max_ = 757 was obtained after *t*_x_ = 52.3 s. After *t*_x_ = 53.1 s, a cocktail of 50 μM BAPTA AM and 10 μM EGTA was injected into the chamber, and the imaging was performed for another 10 min or until the fluorescent signal dropped to the minimum *F*^P^_min_ = 126. Scale bar = 30 μm. The color bar on the right corresponds to the normalized calcium dependent intensity level. **b** Histogram of Cal-520 intensity over the RBC population at selected timepoints. The Gaussian distribution was fitted to the histogram to obtain the mean intensity of the population. **(c)** Normalized fluorescence intensity of labeled RBCs population over 150 s. Key events (i.e. addition of A23187 and BAPTA AM + EGTA) are indicated. Measured *F*^p^_rest_, *F*^p^_max_ and *F*^p^_min_ were 292, 757 and 126, respectively. **d** Boxplots of normalized *F*_max_ intensity of the RBC under different aspiration conditions after the addition of A23187. No significant difference in *F*_max_ was observed amongst cells resting on the chamber surface, being aspirated by the water manometer or the pneumatic HSPC. *n.s.* not significant, assessed by two-tailed Student’s *t*-test

From a user-friendly perspective, determining the Cal-520 dynamic range by adding A23187 and BAPTA AM + EGTA from one aspirated cell to another was tedious and labor-intensive. We, therefore, examined whether obtaining the average *F*_min_ and *F*_max_ from a population of RBCs in the field of view during imaging (*n* ≥ 20) is valid to serve as a once-for-all and efficient alternative approach. First, it is important to validate that the dynamic range obtained from the resting population is identical to the dynamic range obtained from the aspirated single cells. Thus, we pre-loaded a population of RBCs with Cal-520 AM, injected them into the cell chamber and waited until they settled to the bottom of the chamber. Similar to the single-cell determination, frames were initially captured to indicate the *F*^p^_rest_ of cells (Fig. 5a, 1st column), which was used as a reference to validate the measured dynamic range. Then, *F*^p^_max_ and *F*^p^_min_ of the RBC population were obtained in the same way as aspirated cells. After collecting images under these conditions, histograms of measurements corresponding to each condition were built (Fig. 5b, bottom) and the mean of the histograms can be obtained (*F*^p^_max_ = 757 and *F*^p^_min_ = 126) as the population average calibration.

Moreover, the fluorescence intensity of the cell population was also plotted against elapsed time to show the calcium changes over the entire calibration process (Fig. 5c). As indicated, after adding the A23187 and CaCl_2_, the mean Cal-520 intensity amongst the population of RBCs increased by 2.6-fold relative to the resting state (757 a.u. vs. 292 a.u.; Table 1). On the other hand, the fluorescence intensity slowly dropped to the background as expected after the calcium chelating cocktail was added. Compared to the calibration outcome from the single-cell aspiration assays (i.e. ∆*p* = − 1.84 mmHg and − 2.94 mmHg from the water manometer; ∆*p* = -5 mmHg and − 25 mmHg from the pneumatic HSPC), the fluorescence intensity in all tested RBCs increased by 2.5 to 3-fold after A23187 was added (Fig. 5d), and the intensity decayed to the background level under all conditions after the BAPTA AM + EGTA cocktail was added. Taken together, the Cal-520 dynamic range determination from the RBC population is valid to serve as an alternative once-for-all calibration and is transferrible to the single-cell aspiration settings.

### Quantitation of RBC cytosolic calcium concentration

**Fig. 6:**
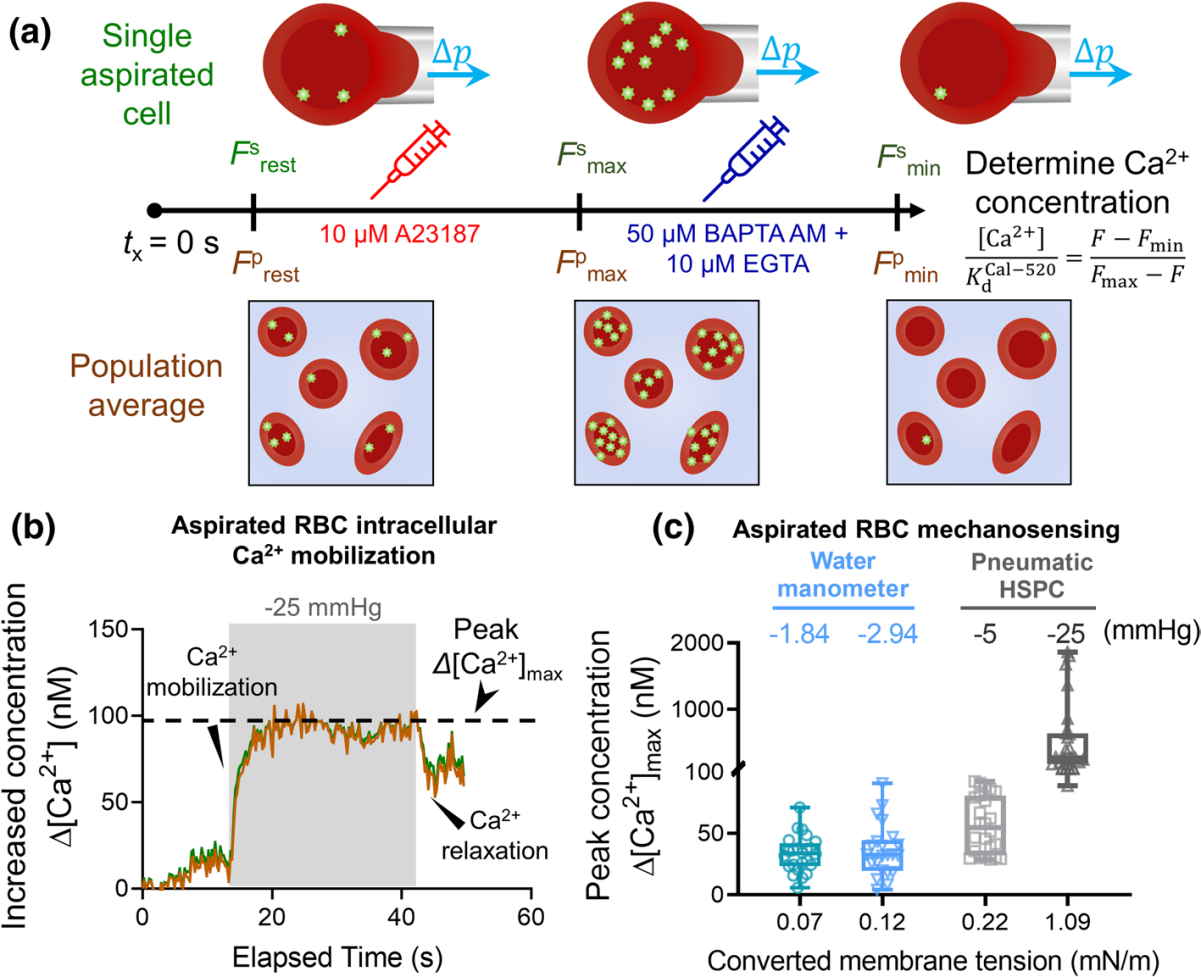
Quantification of real-time intracellular calcium concentration of the aspirated RBC. **a** Schematic of quantification of Ca^2+^ mobilization. Using the Eq. (3), two different calibrations obtained in previous sections were implemented to derive the live time *∆*[Ca^2+^] (nM) of the aspirated RBC. **b** Representative line graph of Increased concentration *∆*[Ca^2+^] (nM) vs. Elapsed time (s) derived by different calibration methods over the *∆p* = − 25 mmHg aspiration. The real-time [Ca^2+^] from both populations average and single-cell dynamic range calibrations shows slight difference. A significant *∆*[Ca^2+^] was shown when the negative pressure was applied to the RBC, where the peak *∆*[Ca^2+^]_max_ is ~ 100 nM. **c** Boxplots of calcium mobilization under stimulation of different membrane tension. The membrane tension of the RBC was evaluated by the law of Laplace shown in Eq. (2). At 1.09 mN/m, up to 2000 nM peak increase in the [Ca^2+^], *∆*[Ca^2+^]_max_ was noted. Such a large value can be due to the fluorescence intensity of the cell approaching the upper limit of the dynamic range, which may cause the calculation to become inaccurate

To gain more mechanobiological insights into the RBC mechanosensing quantitatively, we further calculated the real-time intracellular calcium concentration [Ca^2+^] using the following equation (Szydzik et al. 2019):

3 $$\frac{[{Ca}^{2+}]}{{K}_{d}^{Cal-520}}= \frac{F-{F}_{\mathrm{min}}}{{F}_{\mathrm{max}}-F}$$

where *K*_d_^Cal−520^ (Table 1) of the Cal-520 AM probe is 320 nM (Lock et al. 2015), *F* was the instantly measured fluorescence intensity of the aspirated RBC, *F*_max_ = 757 was the maximum mean fluorescence intensity amongst cell population obtained previously, and *F*_min_ = 126 was the minimum mean fluorescence intensity (Fig. 6a). Alternatively, the single-cell maximum, *F*^s^_max_ = 821 (Table 1), and minimum, *F*^s^_min_ = 129 were also implemented in calcium concentration calculation. We then calculated calcium concentrations using both calibration methods at different timepoints, where the increase concentration, ∆[Ca^2+^] (nM) of the aspirated RBC was plotted against the elapsed time *t*_x_ (s) (Fig. 6b). To this end, identical *∆*[Ca^2+^] is derived by both population average and single aspirated cell calibration, since both *F*_max_ reflected a consistent magnitude of increase amongst resting and different aspiration conditions (two-to-threefold, cf Fig. 5d). This result further demonstrated the validity of implementing population calibration in deriving *∆*[Ca^2+^] in a single aspirated RBC.

Furthermore, when the membrane tension of the aspirated RBC was evaluated by Eq. (2), the peak intracellular calcium concentration, *∆*[Ca^2+^]_max_ was ~ 30 nM and ~ 100 nM at 0.22 and 1.09 mN/m, respectively. It is also worth to note that some RBCs with 1.09 mN/m membrane tension had much larger *∆*[Ca^2+^]_max_ up to the low micromolar range (~ 2000 nM; Fig. 6c). These results clearly indicate that an increasing aspiration can trigger the activation of a Ca^2+^ permeable mechanosensitive channel on the RBC membrane, causing the [Ca^2+^] elevation by a hundred times higher than the normal physiological condition (Fig. 6c).

## Discussion

Intracellular calcium concentration plays a vital role in RBC physiology and pathophysiology. The calcium signaling is associated with multiple physiological processes (Bogdanova et al. 2013; Hertz et al. 2017); however, the detailed mechanism of how mechanical stimulation triggers this signaling pathway remains unclear. Thus, it is crucial to develop assays that are capable of visualizing calcium mobilization and simultaneously quantifying live-time calcium levels in RBCs under controlled mechanical stimulation. In this study, we developed a micropipette assay coupled with fluorescence imaging using a intensiometric calcium probe Cal-520 AM, which can be used to characterize and quantify the effect of aspiration force on RBC calcium regulation.

Two different aspiration apparatuses, the homemade water manometer and the pneumatic HSPC were implemented, respectively, to aspirate the RBC, which display superiorities in different aspects. It has been demonstrated that − 5 mmHg is a threshold pressure that triggers a detectable increase in the fluorescence intensity of an aspirated RBC (Cahalan et al. 2015). In comparison, limited by its working distance, the strongest pressure that can be delivered by our water manometer is *∆*p = − 2.94–0 mmHg. However, with the benefit of the higher signal-to-noise ratio of Cal-520 AM (Lock et al. 2015), we are able to detect and quantify subtle increase in the Cal-520 intensity when gentle pressure was applied (Fig. 3b and c). Nevertheless, our micropipette aspiration assay using the water manometer can minimize the pre-activation of the RBC. On the other hand, the pneumatic HSPC generates a much higher aspiration pressure (− 200–0 mmHg, ± 1 mmHg) than the water manometer. Hereby, it results in a much stonger calcium mobilization in the RBC than when the water manometer is used to aspirate the cell. By implementing the pneumatic HSPC, our fluorescence-coupled micropipette aspiration assay becomes a powerful tool to characterize the RBC calcium mobilization in response to precisely controlled mechanical stimuli. This system allows to characterize the activation mechanism of Ca^2+^ permeable mechanosensitive ion channels, such as Piezo1, and investigate the mechanotransduction pathways that influence the physiological functions of RBCs.

Operational wise, multiple critical factors determine the success and application of a fluorescence-coupled micropipette assay. First, both incubation time and continuous mixing of the Cal-520 AM dye during the loading process are essential for sufficient dye uptake. It is thus ideal to incubate RBCs and the dye in a tube on a vertical spinner. Second, given that high dose of the solvent of the probe, DMSO, is toxic for living cells, DMSO dilution higher than 1:250 is recommended when loading the Cal-520 AM. Third, to get the best quality of cell segmentation, the thresholding algorithm during calibration needs to be properly selected. Imaris, an integrated commercialized software, can automatically determine the optimal thresholding algorithm itself for image processing and data analysis. Furthermore, when operating the micropipette aspiration, it is critical to level down the tip to the bottom surface, where cells rest. This ensures that the vertical position of the cell will not fluctuate out of the focus, otherwise the measurements of Cal-520 intensity are inaccurate.

Compared to other methods, such as the tonicity assay, micropipette-based techniques are easier to assess and quantify mechanical stimuli and the mechanical properties of cells (Wang et al. 2022b), such as membrane tension using relevant physical models (Fig. 1b) (Mierke 2021). We also provide suggestions on algorithm selection to improve the quality of RBC segmentation in fluorescence images. This advance enables measurements of calcium signaling under fine-tuned mechanical stimuli and improves the efficacy. More importantly, micropipette assays have the potential to be combined with emerging dynamic force spectroscopy methods, such as the biomembrane force probe (Chen et al. 2015) and optical tweezers (Wang et al. 2022a), and be used to measure other types of forces, including tension, compression and shear stress, to study mechanosensing.

## Data Availability

Data is available on request from the corresponding author. The data are not publicly available due to privacy or ethical restriction.
